# Community perception on biomedical research: A case study of malariometric survey in Korogwe District, Tanga Region, Tanzania

**DOI:** 10.1186/1471-2458-14-385

**Published:** 2014-04-22

**Authors:** Isolide S Massawe, John P Lusingu, Rachel N Manongi

**Affiliations:** 1 National Institute for Medical Research, Tanga Centre, Tanga, Tanzania; 2 Kilimanjaro Christian Medical University Collage, Moshi, Tanzania

**Keywords:** Community, Perception, Biomedical research, Experience, Challenges, Community owned resource persons, Tanzania

## Abstract

**Background:**

Community perception in biomedical research remains critical in Africa with many participants being driven by different motives*.* The objective of this study was to explore the perceived motives for women or females guardians to volunteer for their children to participate in biomedical research and to explore experiences and challenges faced by Community Owned Resource Persons (CORPs) when mobilizing community members to participate in biomedical research.

**Methods:**

This cross sectional study was conducted in Korogwe district, in north-eastern Tanzania. Qualitative methods combining random and purposive sampling techniques were used for data collection. A randomly selected sample using random table method from the existing list of households in the ward office was used to select participants for Focus Group Discussions (FGDs). A purposive sampling technique was used for In-Depth Interviews (IDIs) with CORPs. Thematic framework analysis was used to analyze the data.

**Results:**

Need for better health services, availability of qualified clinicians, and better access to services provided at the research points were reported as main motives for community members to participate in biomedical research. With regard to experience and challenges faced by CORPs, the main reasons for mothers and guardians not participating in biomedical research were linked to misconception of the malariometric surveys, negative perception of the validity and sensitivity of rapid diagnostic tests, fear of knowing Human Immunodeficiency Virus Infection (HIV)/Acquired Immune Deficiency Syndrome (HIV/AIDS) sero status, and lack of trust for the medical information provided by the CORPs. Challenges reported by CORPs included lack ofawareness of malariometric surveys among participants, time consumption in mobilization of the community, difficulties in identifying individual results, and family responsibilities.

**Conclusion:**

This study has shown that majority of community members had positive perceptions of the about malariometric surveys services provided. The availability of free health services was the major determining factor for community members’ participation in malariometric surveys. CORPs are instrumental in mobilizing community members participation during malariometric surveys, despite their experiences and the challenges they face.

## Background

Participation in biomedical research requires informed consent from potential study participants [1,2]. Recruitment of study participants is the most challenging exercise in biomedical research due to ethical requirements and other logistical issues [3]. There has been an increase in children participating in biomedical research in order to improve health and to ensure best treatment is available to children [4]. However, community members may have negative perceptions of participating in biomedical research involving children for a number of reasons.

Well designed biomedical research can make vital contributions to advancing medical knowledge, improving treatment, care and quality of life. Several studies have been conducted to identify factors that may or will motivate parents to consent for their children to participate in biomedical research. A recent study in Australia showed that parents were able to balance risks and benefits when deciding whether their children should participate in biomedical research [5]. The perceived risks included potential side effects such as rashes, being randomized to ineffective treatments and the inconvenience of participation. A study done in the United Stated of America (USA) revealed that most parents were afraid of their children “being treated as guinea pigs”, but were willing to allow their children to participate in research if asked by their own doctors [6]. One of the most important factors for biomedical research to succeed in recruiting community members including children is for potential participants to be willing to enroll [4,7]. Decision to participate in biomedical research depends on several factors such as perceived benefits like free treatment, suffering from the disease under investigation, monetary reimbursement and altruism (sacrificing something for someone other than the self) [1]. Apart from individual gains some people may volunteer to participate in biomedical in order to contribute to science or improve health of others or having interest in goals of the study, meeting other people or just out of curiosity [8].

The willingness to participate may also depend on the nature of engagement between researchers and the researched community, which can help to create an environment that is conducive for smooth research activities and enhancing a sense of research ownership [9]. However, some participants decline to participate in biomedical research due to fear of potential side effects of the intervention or invasive procedures like repeated blood draws [10], lack of decision making in the family, religious beliefs, mistrust of trial organizations, concerns about efficacy and safety of the investigated product and loss of confidentiality [11]. Perceived benefits include the offer of hope, better care of their children, the opportunity to access new treatments, being attended to by healthcare professionals, access to health information, meeting others in similar circumstances, and helping others.

In Northern India a study involving drawing of blood for testing malaria in children showed that some parents were less willing to allow their children to participate than they were themselves because they were afraid of unknown side effects while others believed that their children had not enough blood [8]. Participation in medical research is also influenced by decision making power in the family and society. Pressure from village leaders, the research team, village elders and spouses has been reported to influence participation in research [10,12,13].

On the other hand, community mobilization to increase participation in the study has been a difficult task. A study done at Kilifi in Kenyan Coastal showed that at local community level, one potential channel linked to success in enrolment of participants is the use of Community Health Workers (CHWs) [14]; these are called CORPs in other localities. CHWs are selected by community members, trained to carry out certain health care functions, are answerable to communities for their activities and are supported by the health care system [15]. They have played an important role in health care delivery in many developing country settings, by filling in service provision gaps where more skilled personnel are not available. They have helped to broaden health care access and coverage in remote areas, have contributed to attainment of Millennium Development Goals such as childhood immunization, and serve as a bridge between professional health care staff and communities.

In Tanzania a study done by Mubi reported that CHWs were important personnel for extending the availability of antimalarial treatment to households [16]. These CHWs are community members, usually two in each village (male and female), who have been identified by village health committees to deal with health issues in their communities. They are involved in mobilizing communities for participation in biomedical research and assist health workers in health facilities.

In Korogwe, candidates were selected for the posts of CORPs based on criteria at least primary/secondary education and most importantly good relationship with community members, and have been used for the management of uncomplicated malaria using first- line anti-malarial drugs based on fever as a guide to start treatment [13]. CORPs have been used successfully at community level for the provision of early diagnosis and treatment of malaria and collection of epidemiological data after undergoing minimal training [17]. They are recruited with the support of village leaders of the study villages. CORPs receive a monthly token allowance to compensate for their time. These CORPs have been involved in mobilization and recruitment of participants for malariometric surveys and fever surveillance in the two villages of Kwamasimba and Mkokola were this study was undertaken.

Community participation in biomedical research remains critical because it enables a two way sharing of accurate information and ideas between researchers and researched communities. This is fundamental as it creates a conducive environment and inculcates a sense of research ownership by communities, important for the smooth running of research activities within the community.

The objective of this study was to explore motives of mothers or female guardians to consent for their children to participate in a malariometric survey, and the experiences and challenges that CORPs face during mobilization of community members to participate in malariometric surveys in Korogwe District, Tanga Region. The findings are expected to highlight critical issues to consider when designing research projects in order to achieve the intended enrolment target, and also to understand community perspectives related to participation in biomedical research activities.

## Methods

### Study sites

The study was conducted in Korogwe District in north-eastern Tanzania. Since 2003 to date, malariometric cross-sectional surveys to determine malaria related morbidities and immunity among residents are conducted annually during malaria peak season (April –June). This study was purposively conducted in the two villages of, Kwamasimba and Mkokola involved in these cross-sectional malariometric surveys.

### Study population and design

The targeted populations for this study were mothers or female guardians of children under five. This is derived from the common assumption that women or female guardians in African settings are the ones who take care of the children. Participants were 18 year and above as they were able to consent. Participants were excluded if they were away from the study area during the interview period and if they were seriously sick during the interview.

A purposive sampling technique was used to select villages (Kwamasimba and Mkokola) because biomedical research activities namely malariometric surveys have been conducted in these villages since 2003 to date, thus providing a good base for getting participants who have been exposed to biomedical research. A randomly selected sample using a random table method from the existing list of households in the ward office was used to select participants for Focus Group Discussions (FGDs). The village leaders helped in identifying households with parents or guardians who met the inclusion criteria. Later the participants were invited to FGD according to the pre-arranged FGD schedule. CORPs involved in the provision of basic malaria treatment in the villages were purposely invited to participate in IDIs.

### Data collection

FGDs were used to obtain information about community perceptions of children’s participation in malariometric surveys. Each FGDs involved 8–12 participants. The FGDs were conducted in a quiet environment away from participants’ homes in order to be free from disturbance and interruptions. All FGDs were conducted in a ‘round table’ manner in the participants’ respective villages. Each participant of the FGD was given a unique number during discussion instead of names so as to maintain confidentiality. The interview guide was used with flexibility during the FGDs. The facilitator was keen to probe for more information in order to bring to life the discussions amongst participants. This process continued until participants were satisfied that they had no further information to add. The FGDs took a minimum duration of one hour.

The facilitator facilitated the FGDs while conversation and notes were recorded by the note taker experienced in qualitative research using a tape recorder and also taking written notes. After each FGD both the facilitator and note taker expanded notes immediately before conducting the next FGD. Contradicting issues were carried to another FGD for clarification.

In each selected village all CORPs (two female and two male CORPs) who mobilized the community members to participate in malariometric surveys activities were invited for IDIs. Before the interviews, demographic information on participants was collected and identity numbers were given to participants chronologically.

Data were analysed manually using thematic frame framework analysis. According to Braun and Clarke thematic framework analysis has six main steps, namely familiarization of the data, generating initial codes, searching for themes, reviewing themes, defining and naming themes and producing the report [18]. For the sake of capitalizing on validity and reliability, all collected information in the field was given a code for confidentiality. The field notes were read regularly during and after discussions in order to confirm that all points discussed had been noted accurately. The narratives were read and coded by an experienced independent researcher from Kilimanjaro Christian Medical University College (KCMUCo), themes were then compared, discussed and agreed upon together with the facilitator.

Descriptive indicators [19] were used to present the findings of the study. The following explanation details how the results are presented in percentages: all (100% of respondents); nearly all (80-90% of respondents); a majority (more than 50% of respondents); fewer than half (around 25-45% of respondents); a minority (10-25% of respondents) and a few (less than 10% of respondents). “A” represents participants in the FGDs and “B” represents participants in the IDIs. Subject numbers were given to participants chronologically.

### Ethical considerations

Ethical clearance was granted by Kilimanjaro Christian Medical University College Ethics Committee. Permission to conduct the study was sought from Korogwe District Medical Officer (DMO) and Village Executive Officer (VEO). Informed consent was obtained from all the voluntary nature of participation, both verbally and in writing, before signing a consent form.

### RATS guidelines

The authors confirm that this study adheres to the RATS guidelines on qualitative research.

## Results

### Demographic characteristics of study participants

A total of 70 (aged 20–60 years) women participated in this study, of which 66 participated in FGDs and four participated in IDIs; most participants were residing in rural areas. Among participants, small-scale farming was a common occupation. Those with formal employment included teachers and CORPs . The maximum ages of participants were 60 year and the minimum age was 20 years; with about half of them married.

### Need for health services

Different motives were cited as to why mothers or female guardians participated in the malariometric surveys, most of which were closely related. However there was a clear indication that people participated because they looked at the project as a good opportunity to access health services and better treatment than the standard care available. Quick service was consistently mentioned by nearly all participants as a reason for participation in the surveys as indicated in the narrative below;

“…..You get the service at any time, even during the night by the community health providers from the village for free…” (A61; FGD 07).

A minority of participants agreed that people participate in malariometric surveys because the surveys commonly offers access to health care that is quicker, more comprehensive and more personal as compared to what is available at the conventional healthcare facilities. When visiting the hospital they could spend several hours in the queue and sometimes only to be told that there were no medicines. For example a 41 year old male commented:

“…….Other people participated because of the quick service provided, when a child is ill and needs to get the service he gets it quickly and it is nearer to access unlike going to search these services from a distant health facility….” (B67).

The cost of treatment was cited as another important motive for participation. About half of the CORPs associated participation in the malariometric surveys with offer of free treatment even for other illnesses not related to the study A female participant of 42 year old stated:

“….Due to the service provided, drugs and treatment to the children when they become ill, helps the community to participate and even making my work easier in motivating….” (B70).

### Availability of qualified doctors

Apart from the need for health service, the majority of participants also noted that the availability of qualified doctors contributed to the people’s participation. For example one participant narrated: 

“…I come to participate to know if my child has malaria or not, because when I bring him his weight is taken, then I visit the doctor for blood test and wait for the results to know if he has malaria or not, if he has less blood they also tell us…” (A53; A06; A63; FGD 06, FGD 01, FGD07 respectively).

About half of the participants were eagerly waiting for the surveys so that they could be attended by a qualified health personnel and get free treatment. One of the common narratives from participants was exemplified in this quotation:

“…*They come in large number in the period of the malariometric surveys because there is a team of the specialists coming together with different drugs..*.”*(B69* 35 year old male).

The majority of participants in FGDs stated that malariometric surveys have been able to reduce the number of deaths of children under five years old. This is attributed to the good treatment offered by the doctors to the community during malariometric surveys. The common narrative among the participants was;

“… the coming of this service has to a large extent decreased the rate of children death because we get treatment from doctors….” (A48; FGD05).

Other participants explained that:

“.... Before malariometric surveys the rate of deaths in our community was very high but after malariaometric program the number of deaths due to malaria started to decline…” (A64; A48; FGD 07,FGD 05 respectively).

### Closeness of services

About half of participants in FGDs said that the closeness of services as one of the major reasons to participate in the malariometric surveys. Most participants explained that during surveys you get service nearer and at anytime. For example the participants stated that;

“…..The service is nearer and we are not going to Magunga Hospital (Korogwe District Hospital), also you get this service any time from these health providers (CORPs)…” (A48; A43; FGD 05).

Other participants explained:

“….The expenses has decreased a lot because Magunga Hospital is far, you are obliged to have enough money for transport fare and tea but now we get this service here….” (A58; A17; FGD, 06; FGD 02 respectively).

### Misconception on malariometric surveys

When we explored the hindering factors for women or female guardians to allow their children to participate in biomedical research we found out that there were some misconceptions on malariometric surveys, such as fear of HIV testing and rapid malaria diagnostic test (RDT) sensitivity.

For example a 27 year old participant stated:

“...... I suspect they are hiding something because they are telling me I am malaria negative but probably I might be suffering from something else. Because they deal with malaria only they are unable to tell the truth in case I am suffering from something else….” (A62; FGD 07).

Another participant, 38 year old added:

“…What I think is, when they come for their research it is better they tell us the truth that they will also test for HIV, when they come and a person is tested she should be given her results even if she is suffering from typhoid, they should tell us the truth. It is even not bad to tell us where the blood is taken to and also we should know if they are doing malaria research only? If it is only malaria then they should advise me from not being afraid when a lot of my blood is taken and it is better that they tell the truth if they test for HIV status or something else…..” (A05; FGD 01).

Less than half of the participants, especially in FDGs were afraid and expressed their feelings of the procedure on how the blood was taken. They also feared that more blood would be drawn from their bodies, and failed to understand why blood was taken from the vein instead of finger tips; for them it seemed to be related to witchcraft. For example, participants explained that; 

“…..The problem is, people want the blood to be drawn from the finger tips because only small amount is collected, but from the large vein a lot is collected that’s why they are afraid. When you are doing the test with the CORPs, it (blood) is drawn from the finger tips but when they come (researchers) they draw from the large vein, that’s why people fear… (A19; A17; FGD 02).

Fear of being tested for HIV/AIDS was reported as among the reasons why some people refused to participate in the malariometric surveys. Particularly among men, this was especially related to the fear of knowing their HIV status and also they had little understanding of the different reasons why the blood was drawn from those people participating in the surveys.

“….We also want the fathers to come, because when the announcement is done it is only the mothers and their children who come, the fathers are afraid for HIV test and think that they are going to test for HIV instead of malaria for that reasons they assume that once their women are safe then they are automatically safe also…” (A10; FGD 01).

The two male who participate in IDIs stated;

“…..lack of understanding of why the blood is drawn from the large arm vein, makes the women or female guardians reluctant to participate…. For most of them when the vein is not seen or, when they hear of blood testing, they think it is HIV testing, this makes them afraid….” (B67; B69).

### Decision to participate in malariometric surveys

About half of participants reported that they made the enrolment decisions on their own. When prompted further, the majority reported that it was not difficult because they felt that the services which they received were useful. However, participants said, they were likely to inform their family members/marital partners about their enrolment decisions after they had given their consent. A few made their enrolment decisions after consulting their family members.

“……I involve my family together with my neighbours because this service is important and it came near to us….” (A64; FGD 07).

“….My decision was easier because the service was brought nearer and it was worth to our community instead of going to Magunga hospital…..” (A27; FGD03).

### CORPs’ experience with malariometric surveys

We explored the experience and challenges faced by CORPs in mobilizing community to participate in biomedical research with a special focus on the malariometric surveys. The main objective was to understand the other side of the coin from the perspective of the CORPs who dealt with the participants in terms of seeking their consent, giving treatment and following up those participating in the surveys.

Near all CORPs experienced that it was easy for women or female guardians to take part in malariometric surveys due to the awareness of the benefits which friends and relatives received such as quick service, availability of medical personnel and free drugs. A 41 y ear old male stated: 

“…..Other people participated because of the quick service provided, when a child is ill and needs to get the service he gets it quickly and it is nearer different from going to search from Magunga Hospital ….” (B67).

Another 24 year old female explained:

“….People participate because there are doctors and nurses, so when you are examined and diagnosed with malaria or not or seen with other illnesses you are given drugs, or advised immediately, a person might come seriously as an anaemic then she is taken to the hospital, so when I am found with other illness apart from malaria I get helped ….” (B 68).

As one 35 year old male added:

“….. By the time when researchers arrive for malariometric suvery, the intended or targeted community is more motivated because they know that the researchers are going to check their children’s health and offer them treatment for free…” (B70)

The majority of CORPS expressed that the issue of external exposure is among the hindering factors for mothers or females guardians to allow their children to engage in any research. A 24 year old female CORP explained:

“….Another problem for people is that they do not to understand the environment beyond their villages especially rural environment. Mind you, rural environment is different from the urban environment because a person hasn’t traveled to see other different places, other people were born and have stayed in the same village so when these researchers come it is something strange to them …..” (B68).

A 41 year old male commented:

“…..Others are unable to understand about this malaria study. It is difficult for them to understand, for instance an old woman told me “you are coming to tell me about the study that will not help me because am very old”. But there was a time the same old person had malaria and we gave her the service here…..” (B67).

A 42 year old female added:

“…..my experience is that, majority who attend mostly are mothers and their children. They are the major characters in participation because mothers are the ones who are taking care of the family compared to fathers….” (B70).

During the community mobilization process, about half of CORPs explained how community’s misconception on malariometric surveys was among the reasons that made mothers or female guardians not to participate in such exercise. For example, the community members confused the general malaria test procedures with HIV test procedures and also they did not trust with RDT test results. A 42 year old female stated:

“….. people are ready to participate if the blood is collected from the finger tips, because they say blood drawn from the large vein is a lot and they think that they are tested for HIV …” (B 70).

A 41 year old male commented:

“…also most people don’t participate because it happens that they might feel having malaria- related symptoms and believe that they are definitely suffering from malaria, then when results come out they are malaria negative and you don’t give them any anti-malarial drugs. This really confuses them, so they lose trust on RDT test and ask why should they participate on this program….” (B67).

Mobilization of the community about malariometric surveys posed a challenge. Nearly, all of the CORPs participants reported that mobilization was time consuming particularly because of low literacy and little understanding of biomedical research. For instance, a 24 year old female participant noted:

“…..*It takes time to explain to the participants the purpose of the study and assure the benefits they will get at the end of the day. This will make them to make decisions whether to join or not….” (B 67).*

In other instances, it was difficult to convince somebody who considered themselves as not sick to participate in surveys where blood was being drawn. In many cases, potential participants considered it unnecessary. For example a 42 year old female explained: 

“……Others don’t see the reasons to participate as they see that there is no need because they are not feeling sick ….” (B68).

Other reasons reported for non-participation were related to gender roles and responsibilities. For example, among the men, participation was reported as low because they carried other family responsibilities that were not related to caring directly for the children. In many cases, the women or female guardians said they had the responsibility of looking after the children including when they fell sick. However, the father, as the head of the family would give approval to the mother in order for her to participate. A female 42 year old explained:

“…..Mothers easily participate because they are everything. They ask you lots of questions because each time when the child becomes ill she brings her, she does that because she has the understanding that when I bring her here I will be assisted….” (B70).

## Discussion

This study has highlighted motives for women or female guardians to allow their children to participate in malariometric surveys, which were mainly related to quality of health services provided by the research team, and the experiences and challenges CORPs face during mobilizing community members to participate in malariometric surveys.

From the responses of the participants in this study, it appears that the majority of the participants had chosen to participate in the malariometric surveys looking for better quality health care services commonly missed from public health facilities. This finding is contrary from the study conducted in Malawi where they reported that some participants wanted to participate in order to benefit from the material and monetary incentives like, soap, peanut butter, orange drink, transport money, napkins, mosquito nets, basins and iron tablets that were being given for participation [20,21]. This difference may be attributed to the fact that such incentives were not part of the package for the participants in the current study. In this study the findings revealed that among the reasons for them to participate was availability of doctors which led to better health services provided during malariometric surveys. This observation could be due to the fact that during malariometric surveys doctors were available and this made free malaria tests, medicines and medical advice/consultation easily accessible. Similar to our findings, in another study in the same district it has been reported that people wanted to join in the malaria vaccine project due to the secondary benefits such as free medical care, transport and incentives [21].

The findings of this study revealed appreciation of the availability of medical services close to the community established during malariometric surveys. The appreciation of the closeness could be due to the fact that community had been living too far from the health care facilities similar findings have been reported in Malawi [22]. The study reported that the majority of participants wished to join the project due to the closeness of the services which are near to their houses.

Another significant finding in our study is that the decision process for the women or female guardians to allow their children to participate in malariometric surveys was not difficult because of the awareness of benefits which their relatives, friends and partner had received. The finding are strongly supported by reports from Southwest Nigeria [23] where it was reported that partners and relatives played a limited role in the decision-making process of participants in malaria clinical research. Similar to this, a study done in Uganda reported that majority of mothers in a malaria drug trial in Uganda, solely decided to enrol with only a few needing their husbands’ decision and permission [20]. Also, another study on malaria vaccine trial in Kenya reported female participants involved their spouses at some point in the decision making process [24] while in Gambia it was found that women were free to join the study without their spouses’ permission except where it was perceived that high amounts of blood would be drawn in this case, some fathers were more likely to deny their wives permission to enrol their children [25].

Beside the above findings, the study revealed that there were misconceptions about malariometric surveys particularly rumors regarding the drawing of blood samples from the vein instead of finger tips. Some community members feared to participate in malariometric surveys due to the rumours associated with venous blood samples claiming that the blood was used to test for HIV and not malaria. This perception showed some lack of knowledge among community members with regard to research activities. However after further explanation, most participants knew that the blood sample was used for malaria diagnostic purposes after being educated by the research team. The study in Malawi [20] reported similar findings whereby some community members linked the blood taken by the researchers with business or Satanism, and the intention to suck blood from the children and sell it.

In this study, the CORPs had experienced various challenges during community mobilization. Another significant finding in this study is that, CORPs are still facing challenges in their job due to lack of awareness on malariometric surveys within the community. This may be due to rural environment they are working in which may have lower literacy rates among community members. This is similar to a study done in Canada where it was reported that various socio-demographic factors such as low levels of education hindered community participation in health programs [26].

Another potential challenge this study was that recruitment of study participants was a difficult exercise and time consuming especially when it came to community mobilization in a low literacy setting. A similar observation in the study conducted in France [3] also identified low literacy among community members as a key factor in getting people to participate in biomedical research among other reasons. Furthermore, a similar study conducted in Uganda [27] reported that CORPs were over-emphasizing study benefits during information giving, and that some participants perceived that CORPs were trying to exert some form of pressure on them to participate. In their efforts to encourage people to learn more about the trial, CORPs sometimes faced hostility, especially from community members who were not keen to participate, or at other times when community members expected CORPs to enrol their own eligible children but did not.

The majority of participants reported that other reasons for non-participation were related to gender roles and responsibilities. This finding is supported by a similar study [25] which reported that there were various gender dimensions during assessments of potential risks and benefits prior to consenting for the trial. For instance, some fathers were more likely to deny their wives permission to enroll their children due to family responsibilities such as agricultural activities among others. In other instances, permission to enrol would be granted if the trial was perceived to fill a father’s role of financing health care.

## Conclusion

This study has shown that the majority of community members held positive perceptions of malariometric surveys and the services they provided. The availability of free health services was the major determining factor for community members’ and their children’s participation in biomedical research. Fear of using venous blood to test for HIV remained a significant barrier for participants to join biomedical research studies. CORPs are instrumental in mobilizing community members participation during malariometric surveys, despite the challenges they face.

### Study limitations

This study has some limitations, including biased FGDs to mothers or female guardians only, without including fathers or men guardians who play a key role in decision-making in a family.

The results are applicable only to the two villages in Korogwe where malariometric survey has been conducted and hence cannot be generalized to whole district, region or country or to other population.

## Competing interests

The authors declare that they have no competing interests.

## Authors’ contributions

IM conceptualized, designed the study, participated in data collection and analysis and drafting the manuscript. JL participated in designing the study, supervised field work, and revised the manuscript. RM participated in planning, design study, analysed the data and revising the manuscript. All authors read and approved the final manuscript.

## Pre-publication history

The pre-publication history for this paper can be accessed here:

http://www.biomedcentral.com/1471-2458/14/385/prepub

## References

[B1] Nappo S Iafrate G Sanchez Z Motives for participating in a clinical research trial: a pilot study in Brazil BMC Public Health 2013 13 1 19 10.1186/1471-2458-13-19 23302375 PMC3554489

[B2] Fisher HR Why do parents enrol their children in research: a narrative synthesis J Med Ethics 2011 37 9 544 551 10.1136/jme.2010.040220 21478415

[B3] Vanhelst J Hardy L Bert D Duhem S Coopman S Libersa C Deplague D Gottrand F Bengin L Effect of child health status on parents’ allowing children to participate in pediatric research BMC Med Ethics 2013 14 7 10.1186/1472-6939-14-7 23414421 PMC3582492

[B4] Fleming J Boeck T Involving children and young people in health and social care research 2012 2 park square,Milton park,Abingdon, Oxon, OX14 4RN: Routledge

[B5] Caldwell PH Butow PN Craig JC Parents’ attitudes to children’s participation in randomized controlled trials J Pediatr 2003 142 5 554 559 10.1067/mpd.2003.192 12756389

[B6] Svensson K Ram¡rez OF Peres F Barnett M Claudio L Socioeconomic determinants associated with willingness to participate in medical research among a diverse population Contemp Clin Trials 2012 33 6 1197 1205 10.1016/j.cct.2012.07.014 22885788 PMC3515640

[B7] Close S Smaldone A Fennoy I Reame N Grey M Using information technology and social networking for recruitment of research participants: experience from an exploratory study of pediatric Klinefelter syndrome J Med Internet Res 2013 15 3 e48 10.2196/jmir.2286 23512442 PMC3636115

[B8] Stunkel L Grady C More than the money: a review of the literature examining healthy volunteer motivations Contemp Clin Trials 2011 32 342 352 10.1016/j.cct.2010.12.003 21146635 PMC4943215

[B9] Nyika A Chilengi R Ishengoma D Mtenga S Thera MA Sissoko MS Lusingu J Tiono AB Doumbo O Siriwa SB Engaging diverse communities participating in clinical trials: case examples from across Africa Malar J 2010 9 1 86 10.1186/1475-2875-9-86 20346126 PMC2907873

[B10] DeCosta A Douza N Krishnan S Chhabra MS Shihaam I Goswami K Community based trials and informed consent in rural north India J Med Ethics 2004 30 3 318 323 10.1136/jme.2002.001065 15173372 PMC1733858

[B11] Shah JY Phadtare A Rajgor D Vaghasia M Pradhan S Zelko H Pietrobon R What leads Indians to participate in clinical trials? A meta-analysis of qualitative studies PLoS One 2010 5 5 e10730 10.1371/journal.pone.0010730 20505754 PMC2873955

[B12] Mandava A The quality of informed consent: mapping the landscape: a review of empirical data, from developing and developed countries J Med Ethics 2012 38 6 356 365 10.1136/medethics-2011-100178 22313664 PMC4825806

[B13] Rutta AS Using community-owned resource persons to provide early diagnosis and treatment and estimate malaria burden at community level in north-eastern Tanzania Malar J 2012 11 1 1 8 10.1186/1475-2875-11-1 22554149 PMC3517357

[B14] Angwenyi V Working with community health workers as volunteers in a vaccine trial: practical and ethical experiences and implications Develop World Bioeth 2013 13 1 38 47 10.1111/dewb.12015 PMC3662994 23521823

[B15] Marsh V Beginning community engagement at a busy biomedical research programme: experiences from the KEMRI CGMRC-Wellcome Trust Research Programme, Kilifi, Kenya Bull World Health Organ 2008 86 5 381 389 10.2471/BLT.07.048462 18375028 PMC2682178

[B16] Mubi M Malaria rapid testing by community health workers is effective and safe for targeting malaria treatment: randomised cross-over trial in Tanzania PLoS One 2011 6 7 e19753 10.1371/journal.pone.0019753 21750697 PMC3130036

[B17] Hopkins H Talisuna A Whitty CJ Staedke SG Impact of home-based management of malaria on health outcomes in Africa: a systematic review of the evidence Malar J 2007 6 134 10.1186/1475-2875-6-134 17922916 PMC2170444

[B18] Braun V Clarke V Using thematic analysis in psychology Qual Res Psychol 2006 3 2 77 101 10.1191/1478088706qp063oa

[B19] Bailey RC Muga R Poulussen R Abicht H The acceptability of male circumcision to reduce HIV infections in Nyanza Province, Kenya AIDS Care 2002 14 1 27 40 10.1080/09540120220097919 11798403

[B20] Masiye F Kass N Hyder A Ndebele P Mfutso-Bengo J Why mothers choose to enrol their children in malaria clinical studies and the involvement of relatives in decision making: evidence from Malawi Malawi Med J 2008 20 2 50 56 19537433 10.4314/mmj.v20i2.10957 PMC2748955

[B21] Liheluka EA Lusingu JP Manongi RN Community perceptions on the secondary health benefits established by malaria vaccine trials (RTS, S phase 2 and phase 3) at the Korogwe site in North Eastern Tanzania Malar J 2013 12 1 157 10.1186/1475-2875-12-157 23651535 PMC3651867

[B22] Mfutso-Bengo J Ndebele P Jumbe V Mkunthi M Masiye F Molyneux S Molyneus X Why do individuals agree to enrol in clinical trials? A qualitative study of health research participation in Blantyre, Malawi Malawi Med J 2008 20 2 37 41 19537430 10.4314/mmj.v20i2.10898 PMC3345665

[B23] Osamor PE Kass N Decision making and motivation to participate in biomedical research in southwest Nigeria Develop World Bioeth 2012 12 2 87 95 10.1111/j.1471-8847.2012.00326.x PMC3393803 22708614

[B24] Gikonyo C Bejon P Marsh V Molyneux S Taking social relationships seriously: lessons learned from the informed consent practices of a vaccine trial on the Kenyan Coast Soc Sci Med 2008 67 5 708 720 10.1016/j.socscimed.2008.02.003 18362046 PMC2682177

[B25] Fairhead J Leach M Small M Public engagement with science? Local understandings of a vaccine trial in the Gambia J Biosoc Sci 2006 38 01 103 116 16266443 10.1017/S0021932005000945

[B26] Boyce WF Disadvantaged persons’ participation in health promotion projects: some structural dimensions Soc Sci Med 2001 52 10 1551 1564 10.1016/S0277-9536(00)00268-9 11314851

[B27] Mukanga D Tibenderana JK Kiguli J Pariyo GW Waiswa P Bajunirwe F Kallander K Community acceptability of use of rapid diagnostic tests for malaria by community health workers in Uganda Malar J 2010 9 1 203 10.1186/1475-2875-9-203 20626863 PMC2914066

